# Economic evaluation of bone stimulation modalities: A systematic review of the literature

**DOI:** 10.4103/0019-5413.50852

**Published:** 2009

**Authors:** Melissa L Button, Sheila Sprague, Osama Gharsaa, Sandra LaTouche, Mohit Bhandari

**Affiliations:** Division of Orthopaedic Surgery, Departments of Surgery and Clinical Epidemiology and Biostatistics, McMaster University, Ontario, Canada; 1 Division of Orthopaedic Surgery, Clinical Therapies Division, Smith and Nephew, Canada

**Keywords:** Economic evaluation, cost effectiveness analysis, ultrasound, bone stimulation modalities

## Abstract

Various bone stimulation modalities are commonly used in treatment of fresh fractures and nonunions; however, the effectiveness and efficiency of these modalities remain uncertain. A systematic review of trials evaluating the clinical and economical outcomes of ultrasounds, electrical stimulation, and extracorporeal sound waves on fracture healing was conducted. We searched four electronic databases for economic evaluations that assessed bone stimulation modalities using ultrasound therapy, electrical stimulation, or extracorporeal shock waves. In addition, we searched the references and related articles of eligible studies, and a content expert was contacted. Information on the clinical and economical outcomes of patients was independently extracted by reviewers. Fourteen studies met the inclusion criteria; therefore, very limited research was found on the cost associated with treatments and the corresponding outcomes. The data available focus primarily on the efficacy of newly introduced treatment methods for bone growth, but failed to incorporate the costs of implementing such treatments. One economic analysis was identified that assessed different treatment paths using ultrasound. A total cost savings of 24–40% per patient occurred when ultrasound was used for fresh fractures and nonunions (grade C recommendation). The results suggest that the ultrasound is a viable alternative for bone stimulation; however, the impacts of the other modalities are left unknown due to the lack of research available. Methodological limitations leave the overall economic and clinical impact of these modalities uncertain. Large, prospective, randomized controlled trials that include cost-effectiveness analyses are needed to further define the clinical effectiveness and financial burden associated with bone stimulation modalities.

## INTRODUCTION

As the cost of health care continues to rise, health care administrators must take the cost of different treatment options for patients into consideration. Fractures are a common and widespread problem, and a common complication following a fracture is a nonunion. There are many bone treatment options available for patients suffering from fractures and the associated complications to stimulate bone growth. However, with the need to consider the economic capabilities of hospitals, patients, and surgeons, treatments have to be assessed in terms of their advantages such as improved recovery, efficiency, and effectiveness. Nevertheless, the prevalent concern is whether the extensive cost of the treatment is worth the benefits.

The newer modes of treatment interventions utilized for fractures are ultrasound, electrical stimulation, and extracorporeal shock waves. Each of these treatments may have benefits for the healing rates of fractures. However, each of the interventions also has substantial costs associated with them. For issues such as these, an economic evaluation and analysis of the problem and the treatment options needs to be conducted. An economic evaluation is a method of gathering standardized, quantitative data of the estimated costs of health benefits arising from the available treatment interventions.^1^

We conducted a systematic review of the current literature to locate published economic analyses evaluating the use of ultrasound, electrical stimulation, or extracorporeal shock waves in the treatment of fresh fractures or nonunions.

## ECONOMIC ANALYSES IN ORTHOPEDICS

### Characteristics of economic analyses

An economic analysis serves as a tool for surgeons and hospital administration to evaluate competing strategies of treatment in terms of their efficacy and cost in order to improve patients' outcomes.^1^ In the past, it was rare for an economic analysis to be conducted in surgical research.^2^ However, in recent years with the age of the population rising along with the expenses of health care increasing, more and more economic analyses are being conducted.^2^ To successfully carry out an economic analysis, a defined methodology must be determined to create the most valid and reliable evaluation. Specifically, an economic evaluation must assess both the costs and the outcomes associated with a medical treatment.^2^ A common flaw in the current literature is to examine the costs of a treatment but neglect the efficacy resulting from the treatment. It is essential that costs be analyzed in terms of improvements in patients' outcomes to accurately assess the benefits of a treatment option.^2^

Various sources of economic demands have to be considered. Areas include direct, indirect, and intangible costs.^1^ Direct costs are the finances that are a direct result from the treatment, which includes supplies and workers.^1^ Nonmedical direct costs are costs incurred by patients and their families.^1^ An example of an indirect cost is transportation to the hospital for patients' families. Indirect costs include lost wages and are related to the decrease in workers' productivity as a result of the treatment, whereas intangible costs relate to the loss of functionality in patients and quality of life measures such as pain and suffering.^1^

### Types of economic evaluations

There are various types of economic evaluations. The common types used in the orthopedic literature are cost-minimization analysis, cost-effectiveness analysis, cost-utility analysis, and cost-benefit analysis.

### Cost-minimization analysis

A cost-minimization analysis looks at two treatment options that are equal in terms of medical outcomes; therefore, this method only assesses the difference in relative costs between the options.^2^ This type of analysis is the easiest to conduct and is the most common method utilized in the literature; however, it is not always implemented appropriately.^2^ Frequently, this type of analysis is used when the outcomes following the treatment are not the same.^2^

### Cost-effectiveness analysis

The second type of economic analysis is cost-effectiveness analysis, which examines the cost and efficacy resulting from a treatment option.^2^ From this information, a comparison is made in terms of the amount of extra benefits gained from the additional costs and the result is described in terms of a cost per unit of effect.^2^ A limitation of this method is the difficulty associated with interpreting the results.^2^

### Cost-utility analysis

When results are unclear, a cost-utility analysis can be conducted.^2^ This type of analysis is similar to the cost-effectiveness model; however, the results are expressed in terms of life years adjusted by a measure of cost efficiency determined by the researchers, which is typically the cost per life years.^2^ The benefit of the method is the ease of comparison resulting from a common outcome measure.^2^

### Cost-benefit analysis

The final type of analysis is the cost-benefit analysis.^2^ This type of analysis looks at treatments that result in differential outcomes and assesses multiple effects to allow a direct comparison of a range of alternative treatment methods.^2^ Assumptions of this model are that each treatment is compared to an alternative where nothing is done and that one alternative is superior compared to competing interventions.^2^

### Considerations for economic evaluations

For every type of economic evaluation, there are common considerations and concerns that need to be identified and addressed.^3^ First, the type of study conducted to analyze the economical outcomes needs to be determined.^3^ Ideally, a large multicenter randomized controlled trial comparing the outcomes and costs associated with two or more treatment options prospectively should be conducted.^3^ However, this option is not always feasible due to the limited resources and extensive costs associated with conducting this type of trial^3^. If resources are limited, an alternative approach is to utilize a decision analytic model.^3^ With this model, a literature search of the current evidence regarding medical treatments and costs is conducted and the data obtained from this search are inputted into a decision analysis model.^3^ This requires gaining information from multiple sources and assessing the accumulated literature for relevant estimates of the clinical and economical outcomes of treatment options.^3^ One limitation of this type of study design is if assumptions from the literature are flawed, the results will suffer.^3^ Therefore, this option is not the ideal tactic but it does serve to compliment real-time evaluation measures.^3^

After determining the study design and methodology, the perspective of the evaluation needs to be decided upon.^2^ Many different perspectives can be analyzed including the government perspective, the societal perspective, the payer perspective, or the hospital perspective.^2^ The general recommendation is to use the societal perspective as it examines all the medical costs associated with a treatment but is not limited to these costs alone.^2^

## CURRENT LITERATURE

### Literature search strategy

The authors identified studies, in English, by a systematic search of EMBASE, PubMed, Cochrane, and Medline databases from 1997 to November 29, 2008. Search strategies were tailored for each database in an attempt to maximize the number of relevant articles assessing the cost of the selected bone stimulation modalities: ultrasound, electrical stimulation and extracorporeal shock waves. In addition, all references of articles were checked for relevant citations, as well as, related article listings supplied through the various databases were analyzed to identify additional articles.

### Eligibility criteria

The reviewers applied the eligibility criteria throughout the articles to ensure they complied with the desired methodology and information. Articles were included if they met the following criteria: (1) assessed ultrasound, electrical stimulation, and extracorporeal treatment in terms of economical impact; (2) inclusion of patients presenting with fresh fractures or nonunions; and (3) reporting the effect of ultrasound, electrical stimulation, or extracorporeal shock waves on bone healing. Interim or subset analyses of final trials were excluded.

### Data abstraction

Reviewers extracted data independently from each eligible study. Information on the treatment device, the duration of treatment, patient inclusion and exclusion criteria, patient demographics, and all clinical outcomes were collected.

### Results

**Figure 1:**
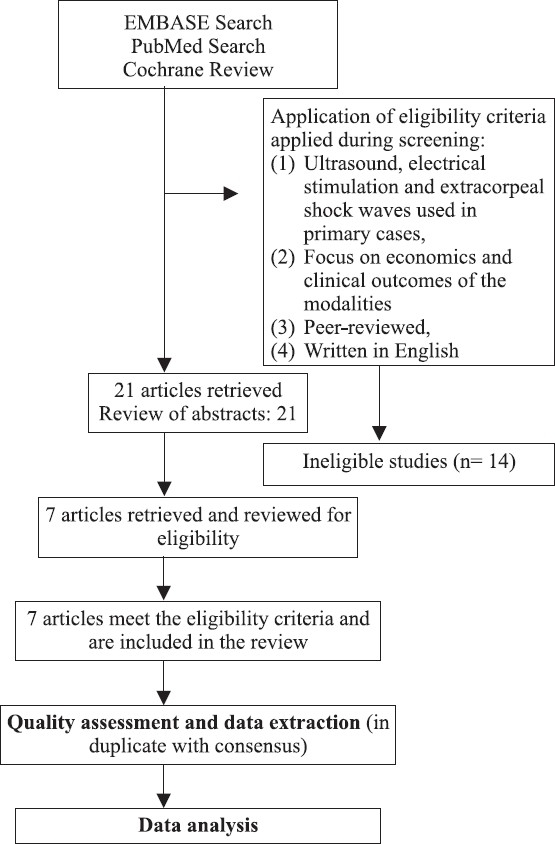
Flow chart of literature search strategy

The search resulted in 1,536 potentially eligible studies; 21 articles were retrieved in full text, and 7 met our inclusion criteria and were included in the final review [Figure 1].

### Description of studies

Through examination of the current literature, it became clear that the impact of ultrasound is the most commonly studied bone stimulation modality. The other methods of bone stimulation, electrical stimulation, and extracorporeal shock waves had extensive literature available on the devices and the resulting outcomes but did not supply any information on the economics or expenses related to the modalities. However, informative data were collected on the clinical and economical outcomes of implementing ultrasounds as a means of bone stimulation in fracture patients for fresh fractures [Table 1] and delayed unions or nonunions [Table 2]. The data were collected from different types of study designs and different types of fracture locations [Table 3].

**Table 1:** Ultrasounds: clinical and economical outcomes for nonunions/delayed unions

Author(s)	Year	Fracture site	Treatment	Currency	Healing rate	Costs		Fractures healed
								
						Type	Amount	
Heckman and Sarasohn- Kahn^4^	1997	Delayed tibial fractures	Ultrasound	US	-	Per case	Conservatively treated with ultrasound cost reduction: $15, 219 Operatively treated with ultrasound cost reduction: $13,259	Delayed union reduced to 6% in the ultrasound group (compared to 36% in placebo)
Siska *et al*.^5^	2008	Fresh fractures	Ultrasound	US	14 months	Per case	$15,000	85% success rate for delayed unions and nonunions
Kanakaris and Giannoudis^6^	2007	Long-bone nonunions	Ultrasound	US	–	Per case	Reduce costs by $13,559.15,219	–
						Avg. inpatient (total charges)	$694
						Avg. Outpatient (total charges)	$21	
						Direct medical costs	$11,333	
Taylor *et al*.^7^	2008	Nonunion tibial fractures	Exogen - ultrasound stimulation	US	Conservative: 79.8%	Cost per patient	$5,488	90.2%
					Conservative and Exogen: 93.6%		$4,704	97.8%
					Surgery: 87.2%		$15,060	87.3%
					Surgery and Exogen: 92.3%		$14,930	93.7%

**Table 2:** Ultrasounds: Clinical and economical outcomes for fresh fractures

Author(s)	Year	Fracture site	Treatment	Currency	Healing rate	Costs			Fractures healed
								
						Type	Amount	
Busse *et al*.^8^	2005	Grade I tibial shaft	Casting and ultrasound	US	96 days	Mean associated costs	$5,017	6.4% (delayed unions/nonunions)
			Casting only	US	140 days	Social perspective costs	$13,266	
						Mean associated costs	$5,312	20% (delayed unions/nonunions)
						Social perspective costs	$17,343	
Rubin *et al*.*^9^	2001	Fresh fractures	Ultrasound		86 days faster than control the group 38% decrease in time to overall (clinical radiographic healing percentage)	Per case	Costs reduced by $13,000–15,000 (including ultrasound therapy)	Only 2 of 33 fractures went on to delayed union (36%) 12 of 33 of the control group went on to delayed union (6%)
Warden *et al*.*^10^	2000	Tibial diaphyseal and distal radius	Sonic accelerated fracture healing system (SONIC)—diagnostic ultrasound unit	US	Accelerates rate by 1.6 (38%—58–37 days)	Cost per case (conservative)	Costs reduced by $15,000 (40%)	Greater than 90%
					Stimulates union in 80–84% of nonunited tibial fractures	Cost per case (surgical)	Costs reduced by $13,000 (23%)

*Results are based on the study conducted by Heckman and Sarasohn-Kahn.^4^

**Table 3:** Study designs

Reference	Study design	Level of evidence	Modality	Fracture location
Heckman^4^	Retrospective comparison study	III	Ultrasound	Delayed unions
Siska *et al*.^5^	Systematic review	III	Ultrasound	Fresh fractures
Kanakaris *et al*.^6^	Systematic review of economic analyses	III	Ultrasound	Long bone fractures
Taylor *et al*.^7^	Economic analysis	II	Ultrasound	Nonunion tibial fractures
Busse *et al*.^8^	Economic analysis	III	Ultrasound	Grade I tibial shaft
Rubin *et al*.^9^	Systematic review	III	Ultrasound	Fresh fractures
Warden *et al*.^10^	Systematic review	III	Ultrasound	Tibial diaphysis, distal radius

### Clinical and economical outcomes

Studies were found that examined the use of ultrasound as a bone stimulation modality. The literature looked at a range of fresh fractures and nonunions including tibial fractures, distal radius fractures, hip fractures, vertebral fractures, Colles' fractures, and scaphoid fractures. Four studies conducted a comparative study analyzing the effect of ultrasound in combination with conservative and surgical treatments or in comparison with a control group.

### Nonunions

Heckman and Sarasohn-Kahn's article was the only one that generated and completed an economic evaluation of ultrasound as a treatment for fractures. Many of the articles found in the literature search referred to the figures and amounts reported by Heckman and Sarasohn-Kahn as it appears to be the only study that calculates the economics of treating tibia fractures and delayed unions. The authors developed three economic models that analyzed two different treatment paths: operative and conservative.^4^ When comparing the model that utilized ultrasound to the model that did not, a total cost savings of over USD 15,000 occurred per patient (20–40%) when ultrasound was used [Table 4].^4^ Most of this savings was a result of less secondary procedures completed and a reduction in workers' compensation costs.^4^ Part of this reduction in costs resulted from the decrease in the rate of delayed unions; specifically, 6% of the ultrasound treatment group suffered from delayed unions compared to 25% of the treatment group not receiving ultrasound.^4^ The authors also stated that early intervention involving ultrasound could neutralize the detrimental effects smoking can have on the unionization of fractures, as well as, cancel out the impact of strata characteristics on the union rates of patients.^4^ Ultrasound also seemed to benefit the older population suffering from fractures as it reduced overall healing time allowing rehabilitation to occur earlier.^4^ This study suggested that a cost-efficient method of treating tibial fractures is to treat the patient proactively with ultrasound therapy instead of proceeding with the standard care treatments.^4^ Not only does ultrasound reduce the economic strain on patients, but it also reduces other complications and results in an earlier healing time, which helps to improve the functionality of patients.^4^

**Table 4:** Total costs and savings of treatment methods^4^

Treatment path	Conservatively treated	Operatively treated
No ultrasound	$38,465	$58,525
Ultrasound	$23,246	$45,266
Total cost savings		
with ultrasound	$15,219	$13,259

Siska *et al*. reviewed different modalities used for bone stimulation.^5^ The authors state economical outcomes for ultrasound but unfortunately did not supply measures of economic outcomes for electrical stimulation or extracorporeal shock wave treatments.^5^ Further studies need to be conducted to analyze the economical demands of electrical stimulation and extracorporeal shock wave therapies.^5^ Siska *et al*., as well as Kanakaris and Giannoudis *et al*., based their economical reportings on Heckman and Sarasohn-Kahn's study. These writers concluded that ultrasound as a bone stimulation modality could result in cost savings of approximately USD 13,000–15,000 per patient.^4^–^6^

Taylor *et al*. found that the use of Exogen, an ultrasound device, with conservative or surgical treatment resulted in a superior healing rate than a conservative treatment or surgical treatment alone [Table 5].^7^ Ultrasound also benefited patients in terms of healing rate at 6 months and costs per patient.^7^ The model utilized by Taylor *et al*. was based on a Markov structure measuring fracture healing as the primary outcome at 12 months.^7^ Cost per patient was calculated as a composite measure of initial diagnosis, rehabilitation and follow-up care, cost of osteomyelitis, and cost of nonunion surgery.^7^ The costs were obtained through the perspective of the payer.^7^ Overall, for a population at risk of nonunions, the authors found that the use of ultrasound resulted in cost savings of USD 744 per patient and an increase in healing rates of 7.6% with conservative treatment of the fracture.^7^ For surgical treatment of the fracture, the cost savings of using ultrasound was USD 130 per patient and improvement in the healing rate was 6.4%.^7^

**Table 5:** Outcomes of ultrasound versus control group^7^

Type of treatment	Healed at 6 months (remaining percentage of delayed unions)	Cost per patient	Healed at 12 months %
Conservative	79.8	$5,488	90.2
Conservative and			
ultrasound	93.6	$4,704	97.8
Surgery	87.2	$15,060	87.3
Surgery and ultrasound	92.3	$14,930	93.7

### Fresh fractures

Busse *et al*. conducted an economic analysis on the treatment options for closed and open grade I tibial shaft fractures.^8^ The treatment options analyzed included casting, casting with ultrasound, or intramedullary nailing.^8^ Evaluation criteria for effectiveness included time to fracture union, which was measured radiographically, and a decision tree was used to measure clinical alternatives.^8^ Both the societal and governmental perspectives were examined.^8^ The associated cost for the casting option with ultrasound was USD 5,312 using the governmental perspective, which was the highest of all the treatment options.^8^ From the societal perspective, the most efficient option was operative management by reamed intramedullary nailing and casting with ultrasound with cost savings of approximately USD 13,266.^8^ Most of the cost reductions came from the patients' lost time at work.^8^

Rubin *et al*. examined the effects of ultrasound on the healing rates of fractures through a review of the literature.^9^ Rubin *et al*. looked at the study conducted by Heckman and Sarasohn-Kahn examining closed or grade I open tibial fractures and the rate of healing comparing the use of ultrasound to a control group.^9^ The ultrasound group resulted in superior outcomes in every measure [Table 6].^9^ Other measures such as patient compliance and complications related to use were extremely positive for the ultrasound group.^9^ However, Rubin *et al*. did not review any studies that included both clinical and economical outcomes.^9^ Based on the findings from Heckman and Sarasohn-Kahn's study, the authors stated that the use of ultrasound in fractures, particularly with patients at risk for nonunions, could result in an estimated savings of USD 13,000–15,000.^4^–^9^

**Table 6:** Results for treatment options for a sample at risk of nonunions^9^

Treatment	Time to heal (clinically)	Time to heal completely (radiographically and clinically)
Ultrasound	86 ± 5.8 days	96 ± 4.9 days
Control	114 ± 10.4 days	154 ± 13.7 days

Warden *et al*. analyzed the results of using the sonic accelerated fracture healing system (SAFHS) in fresh fractures.^10^ The study examined tibial diaphysis and distal radius fractures.^10^ Through a review of the literature, two randomized controlled studies reported a reduction in healing times, both clinically and radiologically, by 38% with the tibial diaphysis fractures healing 58 days earlier and distal radius fractures healing 37 days earlier.^10^ The authors also found benefits in terms of delayed unions or nonunions.^10^ Nonunion tibial fractures treated with SAFHS resulted in unions in 80–84% of cases.^10^ Ultrasound treatment has other benefits in terms of patient care; specifically, patients are able to use the device at home for a brief period of time and are able to borrow the device from the manufacturer.^10^ However, the clinical benefits may be outweighed by the economical strains for individuals.^10^ Overall, the use of ultrasound devices in fractures can reduce the cost of conservative management options by USD 13,000 per case (23%).^4^–^10^ These results are based on the study conducted by Heckman and Sarasohn-Kahn.

The results from the literature review included analyzing the data that focused on the economical and clinical outcomes for patients using ultrasound as a bone stimulation modality. No economic analyses concentrating on shock waves or electrical stimulation modalities were identified. From the available data, it seems as though ultrasound is a cost-efficient alternative for patients, as well as a viable way to improve their quality of life. By using ultrasound as a treatment for nonunions, costs per patient were reduced by USD 130 for patients receiving the surgical treatment, USD 744 for patients receiving the conservative treatment, and USD 15,000 for overall patient costs when compared to patients receiving other modalities as treatment.^4^,^7^ Similar results were found for patients receiving ultrasound as a treatment option for fresh fractures. Costs savings ranged from USD 13,000 to 15,000 per patient.^4^,^9^,^10^

## DISCUSSION

We conducted a systematic review of the current literature to identify economic analyses evaluating the costs and effectiveness of ultrasound, electrical stimulation, and extracorporeal shock waves in fracture healing and fracture nonunions. We identified seven studies evaluating ultrasound therapy. Unfortunately, we did not identify any articles evaluating electrical stimulation and extracorporeal shock waves. While there is an extensive amount of clinical research evaluating bone stimulation methods, the advantages and disadvantages associated with each treatment, the effect of each treatment on clinical outcomes such as bone growth, fracture union, and functional outcome. However, very limited published research was found on the cost associated with treatments.

The seven studies we included in this review suggest that there is evidence to conclude that ultrasound is a cost-effective option for treating both fresh fractures and nonunions. Many of the included studies were decision analyses, which are based on secondary data and require multiple assumptions to be made. Unfortunately, as mentioned above, we did not identify any economic analyses evaluating electrical stimulation and extracorporeal shock waves.

The few published economic evaluations are prone to methodological limitations. A problem associated with data collection in many of the included studies was the lack of statistics in long-term costs resulting from fracture care. Short-term costs are easily calculated as they primarily consist of direct and indirect hospital costs. These costs are reported directly by the patient and families of the patient and, since the patient is still in hospital care, they can be easily contacted. On the other hand, long-term costs are more difficult to maintain as patients can be lost to follow-up or can pass away. With costs associated with short-term or long-term care of patients, both suffer from inaccuracy of the cost estimates caused by variability. Patient demographics vary greatly, with variability in length of hospital stay, severity of the injury, and accessibility for families to the hospital as well. Each of these measures should be reported in order to assist economic analyst to make the most accurate predictions.^11^

### Limitations of economic evaluations: How to deal?

Economic evaluations provide useful information that assist surgeons, hospital administration, and third-party payers with choices of interventions used for patients but there are limitations associated with these evaluations.^2^ Generalizibility can be a problem if the trial is conducted in a setting that is not common to other hospitals or if inclusion and exclusion criteria are too stringent. The setting used in the study needs to mimic the real-life settings observed in hospitals.^2^ Also, if it is feasible, pooling resulting data from various studies helps to increase the level of generalizability.^3^

There are methods and tools available that help limit the disadvantages associated with economic evaluations. The first method is to conduct a sensitive analysis.^2^ This method attempts to limit the effects of uncertainty by determining the dimensions that are believed to vary and from these results, best-guess estimates are generated based on the most conservative and least conservative estimates.^2^ If the overall result is not greatly affected by the estimates used for a certain variable, the conclusions resulting from the data will be stronger.^2^ The other method available is discounting, which takes into account the timing difference in terms of costs and outcomes associated with timing preferences.^2^ In general, people prefer to receive a benefit earlier and costs in the future.^2^ This preference can affect the results of an economic analysis; therefore, discounting takes this into account and discount costs at an average of 3–5%.^2^

Overall, economic evaluations of treatment options are an essential part of assessing the impact of implementing a certain treatment over other available alternatives.^2^ The evaluations serve as a mechanism to identify the treatment that produces the greatest health outcomes with the funding available.^1^ Health care funding will be an ongoing problem for surgeons and patients but conducting more economic evaluations on a wide range of treatment options will help to determine the superior intervention in terms of cost and efficacy resulting in the most superior outcome financially and functionally.

### Future directions for improvement and conclusions

This review indicates that from an economic standpoint the current literature available concerning bone stimulation modalities for fracture healing is very limited, specifically, concerning electrical stimulation and extracorporeal shock waves. Further studies need to be conducted with a detailed analysis of the cost and clinical outcomes associated with the treatment of both fresh fractures and nonunions. Large, prospective, randomized controlled trials that include economic analyses are needed to compare the clinical effectiveness and financial costs associated with the competing bone stimulation modalities. Also, randomized comparative studies examining the efficacy and costs of each of these modalities versus a placebo would be beneficial to gain information on the economic investment and benefits associated with each modality. The current evidence reports only on the clinical outcomes and often fails to incorporate an economic component. Additionally, decision models assessing the clinical question of which bone stimulation modality results in the most effective clinical outcomes while serving as an economically sound alternative should be developed. However, this approach requires multiple assumptions to be made which may lead to inaccurate estimates of clinical effectiveness and costs. Attention and resources need to be dedicated to evaluating the cost-effectiveness of ultrasound, electrical stimulation, and extracorporeal shock waves in the treatment of both fresh fractures and nonunions to inform orthopedic surgeons, hospital administrators, and patients in selecting the most cost-effective and beneficial treatment option available.

## References

[1] Bozic KJ, Rosenberg AG, Huckman RS, Herndon JH (2003). Economic Evaluation in Orthopaedics. J Bone Joint Surg Am.

[2] Drummond MF, O'Brien BJ, Stoddart GL, Torrance GW (1997). Methods for the economic evaluation of health care programmes.

[3] Tanner S, Sprague S, Jeray K (2008). Users' guide to the orthopaedic literature: What is a cost-effectiveness analysis?. Indian Journal of Orthopaedics.

[4] Heckman JD, Sarasohn-Kahn J (1997). The economics of treating tibia fractures The cost of delayed unions. Bull Hosp Jt Dis.

[5] Siska PA, Gruen GS, Pape HC (2008). External adjuncts to enhance fracture healing: What is the role of ultrasound?. Injury.

[6] Kanakaris NK, Giannoudis PV (2007). The health economics of the treatment of long-bone non-unions. Injury.

[7] Taylor MJ, Chaplin S, Trueman P, Searle R, Posnett J Economic Evaluation of the Use of Exogen for Fresh Fracture of the Tibia in Patients at Risk of Non-Union. ISPOR 9th European Conference, Copenhagen, October 2006.

[8] Busse JW, Bhandari M, Sprague S, Johnson-Masotti AP, Gafni A (2005). An economic analysis of management strategies for closed and open grade I tibial shaft fractures. Acta Orthop.

[9] Rubin C, Bolander M, Ryaby JP, Hadjiargyrou M (2001). The Use of Low-Intensity Ultrasound to Accelerate the Healing of Fractures. J Bone Joint Surg Am.

[10] Warden SJ, Bennell KL, McMeeken JM, Wark JD (2000). Acceleration of Fresh Fracture Repair Using the Sonic Accelerated Fracture Healing System (SAFHS): A Review. Calcif Tissue Int.

[11] Johnell O (1997). The socioeconomic burden of fractures: today and in the 21st century. The American Journal of Medicine.

